# Evaluation of tooth brushing behavior change by social marketing approach among primary students in Qom, Iran: A quasi-experimental controlled study

**DOI:** 10.1371/journal.pone.0206042

**Published:** 2018-10-22

**Authors:** Ali Asghar Habibi, Mahdia Gholami, Ahmad Reza Shamshiri

**Affiliations:** 1 School of Dentistry, Tehran University of Medical Sciences, Tehran, Iran; 2 Department of Community Oral Health, School of Dentistry, Tehran University of Medical Sciences, Tehran, Iran; 3 Research Center of Tooth Caries Prevention, Research Center of Dentistry Sciences, Tehran University of Medical Sciences, Tehran, Iran; Kurdistan University of Medical Sciences, ISLAMIC REPUBLIC OF IRAN

## Abstract

**Objectives:**

Nowadays traditional training methods for promotion of oral health behaviors cannot meet the demand of the society and there is a need for effective new methods. The aim of this study was to evaluate the effect of social marketing approach versus the traditional method on promotion of tooth brushing habits in primary school students of Kahak and Jafariyeh, Qom, Iran.

**Materials and methods:**

This study was conducted in the 2016–2017 academic year. First, the reasons for lack of interest in proper tooth brushing were evaluated. Forgetting and laziness were determined as the most important reasons. According to these results, appropriate intervention tools related to proper tooth brushing habits were designed. Then, the students’ tooth brushing habits were recorded before the intervention. Students in Kahak that were considered non-randomly as intervention group received the designed educational package according to the social marketing approach for one and a half months. In Jafariyeh (control group), the students received training only through pamphlets as traditional method. After the intervention, the tooth brushing habits of the students were recorded. The habits before and after the intervention were compared using statistical tests.

**Results:**

Increased length of tooth brushing to at least two minutes was 28.0% in Kahak and 14.0% in Jafariyeh (P<0.001) and increased frequency of tooth brushing to at least two times per day was 30.5% in Kahak and 11.8% in Jafariyeh (P<0.001). Improvement in tooth brushing habits (at least two minutes twice daily) was 32.9% in Kahak and 13.0% in Jafariyeh (P<0.001).

**Conclusion:**

The use of the social marketing approach is more effective than traditional methods in promoting oral health behaviors.

## Introduction

One of the WHO global goals for oral health objectives 2020 is to decrease the rate of DMFT, especially D (decayed tooth), in subjects under 12 years with particular attention to high-risk groups [1]. This objective cannot be achieved unless oral health principles are observed. According to the recommendations of the American Dental Association, people are required to brush their teeth with a soft-bristled brush twice daily for at least two minutes (at least 30 seconds per each quadrant) [2].

Different models have been proposed to promote oral health. One of these models is social marketing proposed by Kotler and Zaltman in 1971 [3]. Social marketing is the use of commercial marketing principles for analysis, design, implementation, and assessment of programs aiming at affecting voluntary behaviors of the target population to enhance the well-being of people and the society [4]. Social marketing has unique characteristics that are absent in other models. These characteristics include the following: 1. It is based on “behavior” change, 2. It is completely client-centered and follows the client’s needs, 3. It relies on proposing interesting offers to persuade the client to change his/her behavior [5]. Different tools can be employed to organize the activities during social marketing, including the Social Marketing Assessment and Response Tool (SMART) developed by Neiger and Thackeray in 1998. This tool has seven phases including Preliminary planning (Problem identification, goal setting, assessment method), Audience analysis (identification and needs assessment), Channel analysis (identification of appropriate communication channels), Market analysis (identification of supporting factors and competitors for behavior change, market mix principles (4P: product, price, place, promotion)), Development (preparation of intervention tools), Implementation, and Evaluation, in the mentioned sequence [6].

A systematic review in 2010 showed that social marketing principles were necessary and effective in designing the campaigns of oral and dental health [7]. Similarly, oral health promotion campaigns in China and Russian, which were based on the social marketing approach, caused a significant improvement in the frequency of brushing to at least twice per day [8, 9]. Some other studies have also shown the success of the social marketing approach in increasing the awareness and improving the performance of the subjects regarding the necessity of regular oral visits for detection of oral cancer [10, 11]. Moreover, another study in Iran in 2014 showed improved awareness of adults about periodontal diseases following a mass media oral health promotion campaign [12].

Since, children should undertake the responsibility of oral health care at the end of six years and parents have a supervisory role, considering its coincidence with the start of primary school education, oral health interventions in this period can provide a good opportunity to establish oral health behaviors in the children [13].

Health messages can be transmitted through various types of mass media such as film, newspaper, pamphlet, the Internet, etc. [14]. Meanwhile, the pamphlet is yet one of the most popular, simplest and efficient tools for message transmission and oral health education that has been proven to be effective in many studies [15, 16]. Considering the confirmed positive role of the pamphlet in this area, we can use it as a traditional intervention compared to the intervention based on social marketing that is a new approach particularly in the field of oral health, which is less widely used and evaluated.

Considering the importance of tooth brushing in oral health promotion and since no similar study has evaluated the effect of the social marketing approach on the tooth brushing behavior change in the target population, we decided to conduct this study. The aim of this study was to enhance tooth-brushing behavior in terms of its length and frequency in primary school students using the social marketing approach.

## Materials and methods

### Study design

This quasi-experimental controlled trial had a parallel design. The study was conducted in two small cities of Kahak and Jafariyeh with low socio-economic status, located in Qom province, Iran. There are cultural, social and geographic similarities among these two cities. In this study, all primary school students (grade 1 to 6) of Kahak and Jafariyeh in the 2016–2017 academic year were eligible to join the study. Non-cooperation of the students and their parents’ unwillingness were the exclusion criteria. Considering the relatively small number of students in the two cities, sampling was not done and the whole target population entered the census. The students in Kahak were selected non-randomly as intervention group. Data collection was conducted at three stages. First we evaluated reasons of students’ disinterest in tooth brushing by a checklist to design appropriate intervention tools. Second, another checklist including school information (city, school name, grade), demographic characteristics (age, gender, parents’ education) and tooth brushing habits (frequency and length of tooth brushing) were distributed among the participants before the intervention (baseline evaluation). Third, the same check list to the baseline was filled out again after the intervention to evaluate changes in tooth brushing habits. The checklists were completed by students themselves at the forth to the sixth grade and completed by parents in first to third grade students.

#### Intervention group (Kahak)

In this city, intervention was done according to the social marketing approach. The SMART model was used to organize different phases of social marketing in the following order [6, 17]:

**Phase 1: Preliminary planning.** The main problem, to overcome which this study was designed, was lack of interest in tooth brushing with the proper length and frequency. Therefore, the main objective of the study was to improve tooth-brushing habits as at least two times a day for at least two minutes each time. The main method that was used for project assessment was a self-report checklist before and after the intervention and comparison of the checklists.

**Phase 2: Audience analysis.** All the primary school students of the two cities (Three primary schools in Kahak and six primary schools in Jafariyeh) were identified as the target population. To evaluate and prioritize the reasons for students’ irregular or no tooth brushing, from the perspective of parents (for the students in grade first to third) and the perspective of the students in grades forth to sixth, a checklist was provided and its results were used in designing the intervention tools.

**Phase 3: Channel analysis.** Since most of the students’ time was spent at home or school, communication channels to educate the students were defined in these environments. For example, school-related channels included face-to-face training sessions, educational materials that can be installed in the school such as poster. Home-related channels included teaching children by parents, application of oral hygiene kits.

**Phase 4: Market analysis.** In this study, one of the supporting factors was the performance of some teachers that considered a day of the week as the “tooth brush day”. On this day, the teachers asked the students to bring their toothbrushes and brush their teeth under the teacher’s supervision.

One of the identified competitors was the parents’ low level of oral health knowledge in Iran [18]. Another competitor was the common belief among the students that brushing the teeth once a day is enough [19].

The second part of market analysis was implementation of market mix principles or 4P as the following:

1. **Product:** The main product was prevention of tooth decay and the real product was tooth brushing two times a day for two minutes each time.
2. **Price:** The time spent in the class (about 30 minutes), time spent on brushing the teeth and commitment to completing the tooth brushing calendar were among the expenses that the participants met.
3. **Place:** The place that was considered for the intervention was the students’ schools and houses because they were easily accessible.
4. **Promotion:** All the tools that were designed to persuade students to brush their teeth are placed in this group. These tools were planned based on the results of the first checklist.

**Phase 5: Development.** In this study, a sample of the education tools was presented to health authorities of education department and managers of target schools in Kahak and Jafariyeh to collect their comments about the appearance and content of the tools legally and culturally. The intervention tools were approved and no comments were received.

**Phase 6: Implementation.** In this phase, the project was implemented as designed in the previous phases, and interventions were applied in the schools. First, a checklist containing questions on students’ tooth-brushing habits was completed before the intervention. After the checklists were collected, the intervention was continued for one and a half months. At the beginning of the intervention phase, an educational workshop about proper tooth brushing habits was held for the students by a research member (HA). Then intervention tools such as pamphlets, toothbrushes, calendars for recording brushing habits, small hourglasses for checking length of brushing and stickers containing messages about tooth brushing were distributed among the students and the explanation about each tool was given to the participants. Also educational posters were installed in school halls. Content of the poster and the pamphlet was related to importance of oral diseases, role of dental plaque in development of the diseases, importance of oral hygiene and proper tooth brushing habits. In addition, teachers and health educators were asked to check the calendar of recording brushing habits every week and review proper tooth brushing habits with students as reminders. In the third and sixth weeks of the intervention phase, the students who perfectly filled out the calendar were given stationery as a reward.

**Phase 7: Evaluation.** The participants completed the tooth-brushing habits checklist before and after the intervention to evaluate the changes following the intervention.

#### Evaluation of the reasons of disinterest in tooth brushing and designing the intervention

In this phase, of 1720 primary school students (535 in Kahak and 1185 in Jafariyeh), 1178 participants (319 in Kahak, 859 in Jafariyeh) completed the checklist (response rate = 68.4%) containing eight items regarding reasons of disinterest in tooth brushing. These items included “Not having a suitable toothbrush for children”, “Not having a toothbrush due to high prices”, “Lack of information about the necessity of brushing”, “Lack of knowledge about correct Tooth brushing Method”, “Lack of parental support for brushing at home”, “laziness”, “dislike of toothpaste flavor” and “Forgetting”. The respondents could select more than one item simultaneously. The results showed that the most important reasons of students’ disinterest in tooth brushing were forgetting (53.5%) and laziness (30.2%). Other items were selected by less than 10% of the respondents. According to these results, appropriate intervention tools were designed for Kahak to meet these needs. The designed tools were:

1. **Tools appropriate for problem of forgetting**
  - Providing stickers with the motto of the project to remind children of brushing their teeth (the motto was “brush twice a day in morning and night, each time two minutes). Number: 535 (for whole students of Kahak primary schools)
  - Designing a tooth brushing calendar for recording the frequency of tooth brushing. In this calendar there were two tables for each week to mark tooth brushing at mornings and nights. Number: 417 (students eligible for the intervention)
  - Providing stationery with the project motto included pen and notebook. Number: from each one 417 (students eligible for intervention)
  - Providing posters appropriate for the students’ age and exhibiting them in different parts of the school. Number: 15 (for three primary schools of Kahak)
  - Reviewing proper tooth brushing habits during the week and checking the calendar regularly by teachers and health teachers.
2. **Tools appropriate for problem of laziness**
  f) Holding an educational workshop about proper tooth brushing habits by a member of the research team. Number: 1 in each school (at the beginning of project)
  g) Preparing educational pamphlets in two separate parts for students and parents. In the student part, the text was suitable for children. In the parent part, it was asked from the parents to have supervision on brushing habits of their children. Number: 535 (for whole students of Kahak primary schools)
  h) Preparing small hourglasses for students to show the time needed for brushing (two minutes). Also the project motto was printed on them. Number: 417 (students eligible for intervention)
  i) Preparing tooth brushes with special designs for children. Number: 535 (whole students of Kahak primary schools)

#### Control group (Jafariyeh)

The students in Jafariyeh completed the tooth-brushing habits checklist before the intervention (similar to students in Kahak). After collecting the checklists, since this city was considered as the control group, a traditional intervention, i.e. pamphlet that had exactly similar content to the pamphlet distributed in Kahak was applied (Number:1185 for whole students of Jafariyeh primary schools). The pamphlet was distributed between the students and we asked them to read it themselves and their parents. Then, the changes in tooth brushing habits were assessed through completing the same checklist for a second time, after the intervention.

### Outcome variables

We considered three variables including Length of tooth brushing, Frequency of tooth brushing and tooth brushing habit as outcome variables. The target behavior of our study included Length of tooth brushing at least two minutes and Frequency of tooth brushing at least two times a day. The participants who did not undertake at least one of the items correctly (length or frequency) before the intervention but did both correctly after the intervention were regarded as improved cases of tooth brushing habit. The outcomes measured two times (before and after the intervention) with interval of one month and a half between the measurements.

### Statistical analysis

Among the demographic variables, parents' education had missing data and the Little test was used to impute these data. Since significant level was less than 0.001, it characterized that missing data in parental education variable was MAR (missing at random). Afterward missing data insertion was done using the EM algorithm (and by predictors of age, gender, and school). Chi-square test was used to compare two variables of “recommended frequency of tooth brushing” and “recommended length of tooth brushing” between the two groups. Logistic regression was applied to evaluate the effect of possible confounders (gender, grade and parents’ education level) on the correction of tooth brushing behavior among intervention and control groups.

At post-intervention assessment, and with a response rate of 75.5% for intervention group and 68.2% for control group, we tested for patterns in lost samples [20]. For this purpose, we compared demographic characteristics of the respondents and non-respondents using the Chi-square test (Table 1). Since the differences between the two groups were statistically significant in two variables, we did sensitive analysis with worst case scenario in which all lost samples in intervention group considered as "unimproved" and lost samples in control group imputed with same frequency of improvement to intervention group (e.g., 33.7% improvement in frequency, 32.1% improvement in duration and 34.3% improvemnent in both frequency and duration) [21].

To identify the subgroups of students who benefit more from the intervention, the analysis of the subgroups was carried out by logistic regression test with the consideration of the interaction between the independent variables (gender, grade and parents’ education level) with the intervention variable. We analyzed the data using the IBM SPSS statistics version 24 (IBM Corp. Released 2016. IBM SPSS Statistics for Windows, Version 24.0. Armonk, NY: IBM Corp.) and considered P values less than 0.05 significantly.

**Table 1:** Demographic characteristics of the participants before and after the intervention in Kahak and Jafariyeh in 2016.

	Intervention (Kahak)				Control (Jafariyeh)			
Variable	Before the Intervention (N = 417)	After the Intervention(N = 315)	Lost sample (N = 102)	P*	Before the Intervention(N = 901)	After the Intervention(N = 615)	Lost sample (N = 286)	P*
	No. (%)	No. (%)	No. (%)		No. (%)	No. (%)	No. (%)	
**Gender**				0.208				<0.001
Boy	256 (61.4)	188 (59.6)	68 (66.7)		421 (46.7)	218 (35.5)	203 (71)	
Girl	161 (38.6)	127 (40.4)	34 (33.3)		480 (53.3)	397 (64.5)	83 (29)	
**Grade**				0.004				<0.001
First	85(20.4)	63 (20.1)	22 (21.5)		176 (19.5)	131 (21.3)	45 (15.8)	
Second	76 (18.2)	60 (19.1)	16 (15.7)		172 (19.1)	112 (18.2)	60 (20.9)	
Third	73 (17.5)	61 (19.1)	12 (11.8)		140 (15.5)	103 (16.7)	37 (12.9)	
Fourth	56 (13.4)	38 (12.1)	18 (17.7)		158 (17.5)	108 (17.6)	50 (17.5)	
Fifth	67 (16.1)	41 (13.1)	26 (25.4)		111 (12.3)	85 (13.8)	26 (9.1)	
Sixth	60 (14.4)	52 (16.5)	8 (7.9)		144 (16.1)	76 (12.4)	68 (23.8)	
**Father’s Education**				0.177				0.198
Underhigh school diploma	319 (76.5)	246 (78.1)	103 (71.5)		639 (70.9)	427 (69.5)	212 (73.8)	
High school diploma &higher	98 (23.5)	69 (21.9)	29 (28.5)		262 (29.1)	188 (30.5)	74 (26.2)	
**Mother’s Education**				0.725				0.462
Under high school diploma	295 (70.7)	225 (71.4)	70 (69.7)		644 (71.5)	437 (71.1)	207 (73.5)	
High school diploma & higher	122 (29.3)	90 (28.6)	32 (30.3)		257 (28.5)	178 (28.9)	79 (26.5)	

*Chi-square, p<0.05

### Ethical consideration

Participation in this study was voluntary. We obtained informed written consent from parents of all students. For this purpose, there was a section at the beginning of the checklists include explanations onthe study objectives and how the students participated in different phases of the study and asked the parents for their permission. The parents, who agreed to have their child participate in the study, signed the paper. For confidentiality reasons, all checklists were anonymous and coded. The Ethics Committee of Tehran University of Medical Sciences approved this study (ethics code: IR.TUMS.VCR.REC.1395.852). Also this study is registered in Iranian Registry of Clinical Trials (IRCT) with trial number of IRCT2016123131675N1.

## Results

### Pre and post intervention assessment

**Fig 1:**
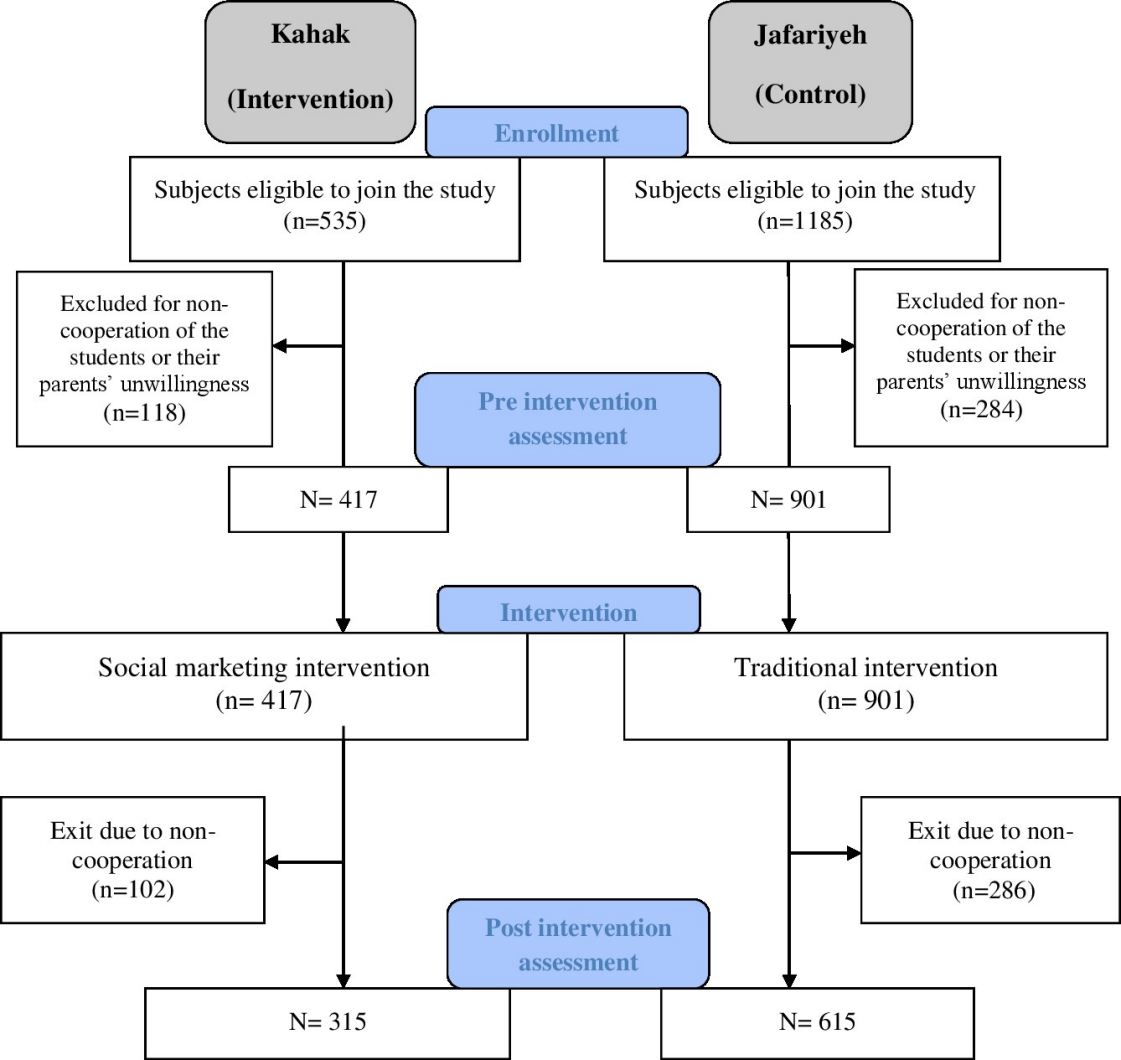
Flow chart of study participants.

Fig 1 shows different phases of the study.

### 1. Demographic characteristics

The demographic characteristics of the students who participated in the pre- and post- intervention phases are presented in Table 1. In relation to educational level of students’ parents, majority of the parents, at the both groups of intervention and control had lower education than high school diploma. So that 76.5% and 70.9% of the fathers at the intervention and control groups had under high school diploma, respectively. These figures were 70.7% and 71.55% for the mothers, correspondingly.

### 2. Results of tooth brushing habits

The results of tooth brushing habits before and after the intervention and their comparison are shown in Table 2. At baseline, most of the students in the intervention and control groups stated that they brush their teeth under parents’ supervision (71.2%, 63.6% respectively). However, these measures got better after the intervention at both groups (81%, 73.5% respectively). The percent of the individuals in the intervention group who mentioned brushing their teeth in two minutes and more improved from 47.7% before the intervention to 74.8% after the intervention. This improvement was also seen in the control group but its value was negligible (42.1% to 48.7%). In addition, 34.5% of the participants of intervention group reported brushing their teeth twice a day or more at baseline and improved to 65.5% after the intervention. These corresponding measures in the control group were 35% and 40.6% that represented a minor improvement in this group.

**Table 2:** Results of assessment of tooth brushing habits of students before and after the intervention in Kahak and Jafariyeh.

Questions	Intervention (Kahak)		Control (Jafariyeh)	
	Before the Intervention	After the Intervention	Before the Intervention	After the Intervention
	No.(%)	No.(%)	No.(%)	No.(%)
**Who brushed the teeth?**				
Student without parents’ supervision	106 (25.4)	56 (17.8)	264 (29.4)	138 (22.5)
Student with parents’ supervision	297 (71.2)	255 (81.0)	571 (63.6)	451 (73.5)
Father or mother	2 (0.5)	2 (0.6)	9 (1.0)	4 (0.7)
Does not brush	12 (2.9)	2 (0.6)	54 (6.0)	21 (3.3)
Total	417 (100)	315 (100)	898 (100)	614 (100)
**Length of tooth brushing**				
Two minues and more	197 (47.7)	234 (74.8)	375 (42.1)	299 (48.7)
Less than two minutes	203 (49.4)	77 (24.6)	461 (51.8)	293 (47.9)
Does not brush	12 (2.9)	2 (0.6)	54 (6.1)	21 (3.4)
Total	412 (100)	313 (100)	890 (100)	613 (100)
**Frequency of tooth brushing**				
Twice a day or more	144 (34.9)	204 (65.2)	313 (35.0)	249 (40.6)
Less than twice a day	257 (62.2)	107 (34.2)	526 (59.0)	343 (55.9)
Does not brush	12 (2.9)	2 (0.6)	54 (6.0)	21 (3.4)
Total	413 (100)	313 (100)	893 (100)	613 (100)

### 3. Changes of tooth brushing habits after the intervention

Table 3 shows participants’ tooth brushing habits improvemet in terms of appropriate length of brushing, appropriate frequency of brushing and proper brushing habits (both the length and frequency of brushing) after the intervention in both groups of intervention and control adjusted for grade, gender and parents’ education.

**Table 3:** Students’ tooth brushing habits improvement after intervention based on social marketing approach in Kahak and traditional intervention in Jafariyeh.

	Behavior change N (%)		OR (95% CI)	p-Value**
	Kahak (n = 315)	Jafariyeh (n = 615)		
Improved tooth brushing behaviour* (twice a day, two minutes each time)	108(34.2)	85(13.8)	3.22 (2.28–4.54)	<0.001
**Length of brushing at least two minutes**	101(32.1)	102(16.6)	2.34 (1.67–3.27)	<0.001
**Frequency of brushing at least two times a day**	106(33.6)	83(13.5)	3.10 (2.19–4.37)	<0.001

OR = odds ratio; CI = confidence intervals.

* The participants who did not undertake at least one of the items correctly (length or frequency) before the intervention but did both correctly after the intervention

** adjusted for gender, grade and parents’ education level

#### 3.1- Tooth brushing habits

In relation with proper tooth brushing habits, 34.2% of the subjects in Kahak (n = 108) and 13.8% of the children in Jafariyeh (n = 85) showed improvement (P<0.001).

#### 3.2- Length of brushing

The length of brushing improved in 32.1% of the students in Kahak (n = 101) and 16.6% of the subjects in Jafariyeh (n = 102) (P<0.001)

#### 3.3- Frequency of brushing

The length of brushing improved in 33.6% of the students in Kahak (n = 106) and 13.5% of the subjects in Jafariyeh (n = 83) (P<0.001)

It was observed that the social marketing approach caused a significant improvement in all three parameters (length, frequency, and both) as compared to the traditional method.

### Sensitivity analysis

As mentioned in statistical method, to estimate the effect of students lost to follow-up on final conclusion we repeated data analysis with all of them in worst case scenario. The worst case scenario was defined as follow: all students lost to follow-up in intervention group considered as "unimproved" and behaviour changes for those lost to follow-up in control group was considered similar to the frequency of improvements of intervention group (e.g., 33.7% improvement in frequency, 32.1% improvement in duration and 34.3% improvemnent in both frequency and duration). For all endpoints, behaviour changes remain significant (improvement in frequency of tooth brushing OR:1.75, 95%CI:1.32–2.32, p-value<0.001; improvement in length of tooth brushing OR = 1.88, 95%CI:1.42–2.50, p-value<0.001; improvement in both frequency and length of tooth brushing OR = 1.54, 95%CI:1.17–2.02, p-value = 0.002).

### Subgroup analysis

In subgroup analysis, the interaction between background variables (gender, grade and parents’ education level) and intervention was not significant except for gender and grade for correcting the length of brushing, indicating a quantitative and qualitative interactions, respectively [22]. Although the intervention was significantly effective in both sexes, it was more effective in male students (OR = 3.36, 95%CI:2.11–5.36 in boys and OR = 1.79, 95%CI:1.13–2.82 in girls). For the effect modification effect of grade on intervention, we found that the intervention was effective in low grades (grades 1–3) and ineffective in higher grades (grades 4–6) (OR = 3.50, 95%CI:2.29–5.36, p-value<0.001 in grades 1–3 and OR = 1.41, 95%CI:0.86–2.31, p-value = 0.17 in grades 4–6). In more details, actually female students in grades 4–6 didn't get benefit from intervention (17.3% vs. 20.3%).

## Discussion

This study reports the effect of the social marketing approach on the tooth brushing behavior change in terms of its length and frequency among primary school students. After the intervention, the social marketing approach caused a significant improvement in length of tooth brushing (at least two minutes), frequency of tooth brushing (at least two times a day), and tooth brushing habits (twice a day, two minutes each time) in comparison with the traditional method.

Through categorization of the target audience and attention to their demands, the social marketing model can design interventions leading to increased participation and acceptance of the desirable behavior (product), while traditional interventions cannot meet the demands of the target population and cause behavior change due to having one solution for all people. For this reason, before designing an intervention, we conducted a research to identify the target population’s needs and their barriers, including the reasons for irregular or no tooth brushing. The results showed that the main reasons were forgetting and laziness.

There are several studies evaluating the effects of social marketing approach in health issues. For example; in a study by Kassegne et al., a social marketing intervention to promote use of oral rehydration salts among caregivers of children under five was relaunched in 2006 in Burundi. Results showed that oral rehydration salts use among caregivers at their children’s last diarrheal episode increased significantly after the intervention. Evaluation analysis showed that a higher level of exposure to the social marketing campaign was associated with greater use of oral rehydration salts and with significant improvements in perceived availability, knowledge of the signs of diarrhea and dehydration, social support, and self-efficacy [23].

In other study by Shams et al, in 2011 in Tehran, a social marketing model to reduce risky driving behaviors among taxi drivers was done. An 8-week educational program was implemented, then risky driving behaviors were assessed by checklists and compared. Results showed that the intervention caused statistically significant reductions in the target behavior in the intervention group compared to the control group [24].

In a study by Rienks et al in 2013 in San Francisco, social marketing techniques were used to develop and implement three campaigns to increase awareness regarding infant mortality disparities and proper infant sleep position and to take action to reduce disparities. This study showed social marketing is an effective tool to increase disparity awareness, especially among groups disproportionately affected by the disparity [25].

This evidence suggests the social marketing approach can produce positive change in health knowledge and behaviour across population. The findings of these studies are in line with our study demonstrated the efficacy of social marketing approach in behavior change.

In the field of oral health, there are also a number of studies to assess the effect of social marketing interventions on oral health promotion. Redmond et al conducted a cluster randomized controlled trials in students with a mean age of 12.1 years from 28 schools of England in 1996. They used an school-based dental health education program to improve knowledge and self-reported behavior. resulted in an improvement in knowledge of dental disease and an increase in the reported duration of brushing. The percentage of the students who reported tooth brushing for more than one minute increased significantly after 12 months of intervention but no significant difference in the frequency of brushing occurred [26], while our intervention resulted in improvement of frequency and length of tooth brushing. Although it is necessary to consider the methodological differences between the two studies and compare the results with caution. In addition, it is important to note that assessment of the results in our study has been done in the short term compared to Redmond study that had a longer follow-up time. However, the strength of our study is related to social marketing approach that resulted in designing the interventions based on the audience's needs.

In a study by Hiiri et al, the rate of brushing twice a day increased significantly after the intervention in children aged 6–15 years [9], which was consistent with our results. This finding can be due to the high similarity of this study to the principles of social marketing since the authors performed a needs assessment to evaluate the current situation before applying the intervention.

Another study that was in line with our results was a campaign conducted in China in 1989–1992 that caused a significant increase in the rate of brushing at least twice a day in children and adults, indicating the success of the campaign [8]. The reasons for this success seem to be related to duration of the campaign, the repetition of interventions and reminders that led to behavior change.

Fallah et al conducted a study in Saveh, Iran to evaluate the effect of an educational intervention for teachers on the students’ oral health but found no significant change in the frequency of tooth brushing defined as at least once a day [27], which is different with the results of our study that used the social marketing approach. Comparison of the results of this study with our findings indicates the needs for interventions based on the social marketing approach.

The external validity (generalizability) of the study was acceptable since no sampling was done and all the target population with maximum heterogeneity was included in the study [28]. We succeeded to implement the intervention components completely for the intervention group. In the present study, systematic blinding was not done, however no bias probably occurred due to the distance was long enough between the two cities. In this situation, it was almost impossible for someone in the control group to receive the intervention of another group.

All students from first to sixth grades were considered in this study. However, considering the possible differences between students' perceptions and tastes in the first to third grades compared to fourth to fifth grades, we suggest that the interventions be designed and implemented separately for the two groups of primary students (first to third and fourth to sixth grades) in future studies.

This study had some limitations. The quasi-experimental design due to non-randomly selection of the intervention group might be the main limitation. In addition, the effect of social marketing approach on tooth brushing habits has been assessed in short term. A loss of 30% that occurred at post-intervention assessment could affect the result validity. However, to estimate the effect of lost sample on final conclusion, we did sensitivity analysis and repeated data analysis with all sample in worst case scenario. The analysis showed tooth brushing improvement remained significant.

Considering the results of this study, the social marketing approach had positive effect to promote tooth brushing habits and caused desirable behavior changes in the target population, although the effect was shown in the short term. More studies with long follow-ups are therefore necessary to assess this improvement. However, findings of the present study suggest policymakers to consider budget for designing programs based on social marketing approach for improving oral health behaviors.

## Supporting information

S1 Fig Flow chart of study participants. (TIF)

S1 Data The SPSS file of study data. (SAV)

S1 Trend The TREND checklist of study. (DOC)

S1 Protocol The trial study protocol (in original language). The English version of it could be found in the link that is uploaded in Data Review URL section of attached files.
(DOCX)
