# Supplementary figures and images for: Evaluation of tooth brushing behavior change by social marketing approach among primary students in Qom, Iran: A quasi-experimental controlled study
Source: PLoS One. 2018 Oct 22;13(10):e0206042. doi: 10.1371/journal.pone.0206042 (PMC6197689; doi:10.1371/journal.pone.0206042)

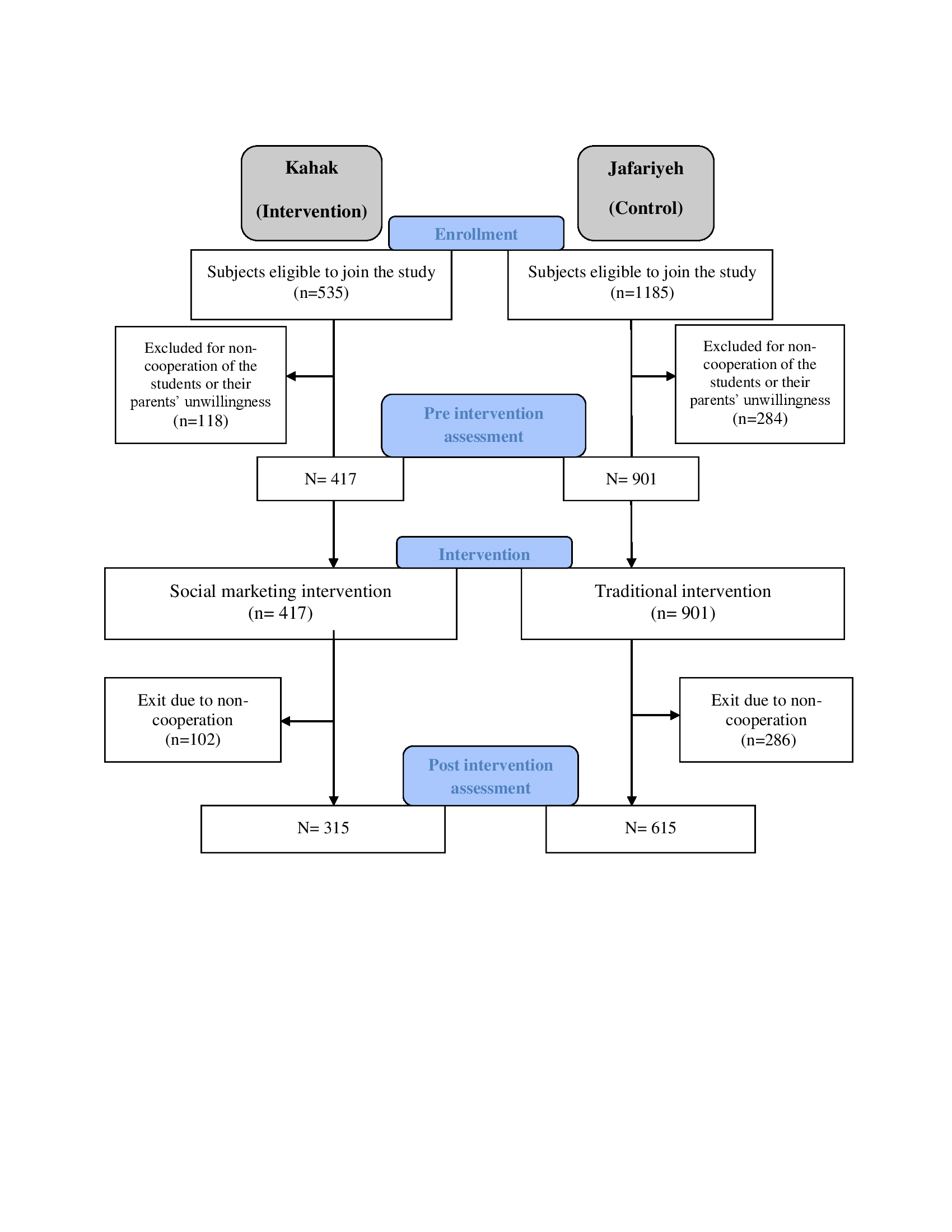

Supplement: S1 Fig — (TIF) [file pone.0206042.s001.tif]
