# Supplementary material for: Evaluation of tooth brushing behavior change by social marketing approach among primary students in Qom, Iran: A quasi-experimental controlled study
Source: PLoS One. 2018 Oct 22;13(10):e0206042. doi: 10.1371/journal.pone.0206042 (PMC6197689; doi:10.1371/journal.pone.0206042)
Supplement: S1 Protocol — The English version of it could be found in the link that is uploaded in Data Review URL section of attached files. (DOCX) [file pone.0206042.s004.docx]

­به نام او

**دانشگاه علوم پزشكي وخدمات بهداشتي درماني تهران**

دانشکده دندانپزشکی

| عنوان طرح:  **بررسی تأثیر دو نوع مداخله آموزشی ( بر اساس اصول بازاریابی اجتماعی و آموزش سنتی) بر افزایش دفعات و مدت زمان مسواک زدن خوداظهار در دانش آموزان مقطع ابتدایی در شهرهای کهک و جعفریه استان قم در سال تحصیلی 96-1395 : یک مطالعه نیمه تجربی شاهددار** |
| --- |

مدير اجرايي طرح : دکتر مهدیا غلامی دانشـكده / مركز تحقيقاتي : دانشکده دندان پزشکی دانشگاه علوم پزشکی تهران گـروه : سلامت دهان و دندانپزشکی اجتماعی

محيط پژوهش : شهر کهک و جعفریه استان قم مدت اجرا : 12 ماه

| خلاصه ضرورت اجرا و اهداف كاربردي طرح :  **امروزه مشاهده روش های سنتی تشویق مردم به رعایت بهداشت دهان و دندان کارایی لازم جهت ایجاد و تغییر رفتار صحیح را ندارند. با پیدایش روش بازاریابی اجتماعی و رویکرد "مشتری محور" آن امید می رود بتوان در جهت تغییر رفتار بهداشتی در مردم و بهبود شاخص های سلامت دهان گام های موثرتری برداشت. در این روش با به کارگیری اصول بازاریابی تجاری در حوزه سلامت تلاش می شود که تا رفتار بهداشتی همانند یک کالا تبلیغ شده و مردم با کسب آگاهی از فواید این رفتار خودشان تشویق شده تا این رفتار را "داوطلبانه" در زندگی خود اجرا کنند و باعث تغییر رفتار آن ها می شود، بدون اینکه رویکرد امری از بالا به پایین وجود داشته باشد. کارایی رویکرد مذکور در حوزه سلامت دهان و دندان و ایجاد تغییر رفتار در جهت ارتقاء سلامت و پیشگیری از بیماریهای این حوزه نشان داده شده است. با این همه از آنجایی که در ایران مطالعه مشابهی بر پایه بازاریابی اجتماعی با هدف تغییر رفتارهای پیشگیرانه در حیطه دهان و دندان موجود نمی باشد ضرورت انجام این مطالعه را نشان می دهد که با توجه به نقش مهم پلاک دندانی در بروز بیماریهای دهان و دندان از جمله پوسیدگی و بیماریهای پریودنتال، تغییر رفتار بهداشتی مسواک زدن از نظر مدت زمان و تعداد دفعات آن بر اساس رویکرد بازاریابی اجتماعی در دانش آموزان مقطع ابتدایی در دو شهر کهک و جعفریه در استان قم مورد مطالعه قرار خواهند گرفت. از آنجاییکه در پایان 6 سالگی، کودک می بایست وظیفه رعایت بهداشت دهان و دندانش را خود بر عهده بگیرد و والدین صرفآ نقش نظارتی خواهند داشت لذا با توجه به انطباق این دوران با آغاز مقطع ابتدایی دانش آموزان، انجام مداخلات دهان و دندان در این دوره، فرصت خوبی جهت نهادینه شدن رفتارهای بهداشتی دهان و دندان و تغییر رفتارهای مثبت در افراد را فراهم می نماید. از طرف دیگر، یکی از ویژگیهای بازاریابی اجتماعی، ایجاد تغییر رفتار در گروهای سنی مختلف می باشد که با در نظر گرفتن دانش آموزان مقطع ابتدایی (از 7 تا 12 سال) با سطوح مختلف توانایی، درک و دانش متفاوت، می توان به ارزیابی کارایی رویکرد بازاریابی اجتماعی در جمعیت مذکور با هدف ارتقاء سلامت دهان و دندان پرداخت.** |
| --- |

| خلاصه روش اجرا:  **در مطالعه حاضر، دانش آموزان مقطع ابتدایی در شهر کهک به عنوان گروه مداخله و در شهر جعفریه به عنوان گروه شاهد در نظر گرفته می شوند. در مرحله پیش از مداخله، عادات مسواک زدن کنونی تمامی دانش آموزان در هر دو گروه، توسط چک لیستی ارزیابی می شود. قابل ذکر است که چک لیست مذکور در پایه اول تا سوم، توسط والدین دانش آموزان و در سایر پایه ها، توسط خود دانش آموزان تکمیل می گردد. سپس در شهر مداخله(کهک) بسته مداخلات آموزشی (شامل توزیع پمفلت، نصب پوستر، بنر و پلاکارد، برگزاری سخنرانی های تعاملی، ارائه مسواک و خمیر دندان دارای لوگوی مطالعه) در ارتباط با مدت زمان و فراوانی مسواک زدن بر مبنای اصول بازاریابی اجتماعی در مدت زمانی حدود یک ماه ارائه شده و در شهر کنترل(جعفریه) صرفا یک روش سنتی آموزشی (پخش پمفلت) انجام می پذیرد.. پس از انجام مداخله، تکمیل چک لیست مجدداً صورت گرفته و بر اساس آنالیزهای آماری نتایج قبل و بعد از مطالعه با هم مقایسه می شود.** |
| --- |

خلاصه هزينه ها

| هزينه پرسنلي | **23،040،000** ريال | هزينه مسافرت | **5،600،000 ريال** |
| --- | --- | --- | --- |
| هزينه آزمايشها و خدمات تخصصي | **20،600،00**ريال | هزينه هاي ديگر | **43،000،000**  ريال |
| هزينه مواد و وسايل مصرفي | **49،820،000 ريال** |  |  |
| هزينه وسايل غير مصرفي | **1،500،000 ريال** | جمع كل | 143،560،000ريال |

# اطلاعات مربوط به عوامل اجرايي طرح:

1. نام و نام خانوادگي طرح دهنده : **دکتر مهدیا غلامی**

رتبه علمي: استادیار

محل خدمت: گروه سلامت دهان و دندانپزشکی اجتماعی

نشاني محل خدمت: امیرآباد شمالی-بالاتر از انرژی اتمی-دانشکده دندانپزشکی

تلفن محل خدمت:88015960

نشاني پست الكترونيك: m_gholami@sina.tums.ac.ir

نشاني يا تلفن براي دسترسي سريع و پيامهاي فوري: 09125472534

درصورتيكه طرح دهنده داراي سمتهاي اجرايي در داخل يا خارج محيط دانشگاه ميباشند جدول زير را تكميل نمايند

| عنوان سمت | نشاني محل كار | تاريخ شروع فعاليت در اين سمت | تلفن محل كار |
| --- | --- | --- | --- |
| عضو هیات علمی گروه سلامت دهان و دندانپزشکی اجتماعی | دانشکده دندانپزشکی، گروه سلامت دهان | سال 93 تا کنون | 88015960 |

درجات علمي و سوابق تحصيلي طرح دهنده به ترتيب از ليسانس به بعد ذكر گردد

| درجه تحصيلي | رشته تحصيلي وتخصصي | دانشگاه يا محل تحصيل | كشور | سال دريافت |
| --- | --- | --- | --- | --- |
| PhD | سلامت دهان و دندانپزشکی اجتماعی | علوم پزشکی تهران | ایران | 92 |
| دکترای عمومی | دندانپزشکی | علوم پزشکی تهران | ایران | 84 |

آيا تا كنون دوره هاي روش تحقيق را گذرانده ايد؟ بلي -*- خير-- در صورت پاسخ مثبت، جدول زير را تكميل فرماييد.

| سطح دوره ونوع آموزشهاي ارائه شده | تاريخ | | محل برگزاري دوره | |  |
| --- | --- | --- | --- | --- | --- |
| کارگاه روش تحقیق مقدماتی | | 1388 | | دانشکده دندانپزشكي تهران | |
| کارگاه روش تحقیق پیشرفته | | 1389 | | دانشکده دندانپزشکی تهران | |
| Research Methods for Postgraduate Dental Students | | 2005 | | دانشکده دندانپزشکی (هلسینکی-فنلاند) | |

سابقه طرحهاي تحقيقاتي كه طرح دهنده قبلا در آن همكاري داشته ويا هم اكنون درحال اجرادارد ، ذكر شود.

| عنوان طرح | محل اجرا | | مدت اجرا | وضعيت طرح | نوع همكاري |
| --- | --- | --- | --- | --- | --- |
| بررسي ميزان رضايت مراجعين كلينيك هاي دندانپزشكي از دندانپزشكان زن ومرد | تهران | یکسال | | اتمام | محقق اصلي |
| بررسی تاثیر رسانه الکترونیک (تلویزیون) در تغییرسطح دانش، نگرش و عملکرد مرتبط با سلامت دهان در بالغین ساکن شهر تهران | تهران | 2 سال | | اتمام | محقق اصلي |
| سنجش عوامل مرتبط با سلامت دهان و دندان زنان باردار | تهران | | 1 سال و نیم | در حال اجرا | همکار طرح |
| بررسی تاثیر فعالیت های فوق برنامه و سرمایه اجتماعی بر میزان افسردگی در دانشجویان دانشکده دندان پزشکی دانشگاه علوم پزشکی تهران در سال 1394 | تهران | | 1 سال | در حال اجرا | همکار طرح |
| تدوین و ارزیابی اجرای «مداخلات سلامت دهان» در برنامه «مدارس مروج سلامت» در دبستانهای استان البرز در سال 1395-1394 | تهران | | 1 سال و نیم | در حال اجرا | محقق اصلی |

1. نام و نام خانوادگي طرح دهنده : **دکتر احمدرضا شمشیری**

رتبه علمي: استادیار

محل خدمت: گروه سلامت دهان و دندانپزشکی اجتماعی

نشاني محل خدمت: امیرآباد شمالی-بالاتر از انرژی اتمی-دانشکده دندانپزشکی

تلفن محل خدمت:88015960

نشاني پست الكترونيك: ar_shamshiri@yahoo.com

نشاني يا تلفن براي دسترسي سريع و پيامهاي فوري: 09125209187

1. نام و نام خانوادگي طرح دهنده : **علی اصغر حبیبی خاوه**

رتبه علمي: دانشجو

محل خدمت: دانشکده دندانپزشکی

نشاني محل خدمت:امیرآباد بعد از انرژی اتمی

تلفن محل خدمت:-

نشاني پست الكترونيك:dreamstar890@gmail.com

نشاني يا تلفن براي دسترسي سريع و پيامهاي فوري: 09196634589

- آيا تا كنون دوره هاي روش تحقيق را گذرانده ايد؟ بلي ■ خير-- در صورت پاسخ مثبت، جدول زير را تكميل فرماييد.

| سطح دوره ونوع آموزشهاي ارائه شده | تاريخ | محل برگزاري دوره |
| --- | --- | --- |
| کارگاه روش تحقیق | ترم هشت | دانشکده دندانپزشکی دانشگاه علوم پزشکی تهران |

1. مشخصات همكاران اصلی طرح :

| رديف | نام و نام خانوادگي | شغل | درجه علمي | نوع همكاري | امضاي همكار |
| --- | --- | --- | --- | --- | --- |
| 1 | محمد تکمار | دانشچو | کارشناسی | توزیع و جمع آوری چک لیست ها-شهر جعفریه |  |
| 2 | رضا رجب زاده | دانشچو | کارشناسی ارشد | توزیع و جمع آوری چک لیست ها-شهر کهک |  |

قسمت دوم - اطلاعات مربوط به طرح پژوهشي

1. **عنوان طرح به فارسي:**

| **بررسی تأثیر دو نوع مداخله آموزشی ( بر اساس اصول بازاریابی اجتماعی و آموزش سنتی) بر افزایش دفعات و مدت زمان مسواک زدن خوداظهار در دانش آموزان مقطع ابتدایی در شهرهای کهک و جعفریه استان قم در سال تحصیلی 96-1395 : یک مطالعه نیمه تجربی شاهددار** |
| --- |

1. **عنوان طرح به انگليسي:**

| **Evaluating the impact of two educational interventions (based on social marketing approach compared to traditional training) to increase self-reported frequency and duration of brushing among the primary students in Kahak and Jafariyeh cities in Qom, Iran in 1395-96: A quasi-experimental controled study** |
| --- |

1. **نوع طرح :**

كاربردي بنيادي بنيادي-كاربردي جامعه نگر(HSR)

1. **بيان مساله و ضرورت اجراي طرح:**

تشویق مردم به ارتقای سلامت دهان و دندان همواره یکی از اهداف فعالین حوزه دندانپزشکی به ویژه بخش دندانپزشکی اجتماعی بوده است. در طی سالیان دراز مدل های گوناگونی جهت دستیابی به این هدف و افزایش مشارکت فعالانه مردم در ارتقای بهداشت دهان و دندان ارائه شده است که اغلب آن ها بر پایه آموزش صرف و توجه به موانع آگاهی و نگرش افراد بوده است. در ارتباط با این مدل ها، در سال های اخیر مقوله جدیدی تحت عنوان بازاریابی اجتماعی وارد عرصه ارتقاء سلامت و سلامت دهان شده است که علی رغم داشتن نقاط مشترک با برنامه های ارتقاء سلامت، دارای امتیازات منحصر به فردی می باشد.

به طور کلی بازاریابی اجتماعی استفاده از قوانین حاکم بر بازاریابی تجاری در حوزه سلامت برای فروختن یک "رفتار" مطلوب اجتماعی به جای کالا به مردم می باشد. در بازاریابی تجاری هدف منفعت زود بازده مالی است در حالی که در بازاریابی اجتماعی هدف تغییر رفتار مردم به سمت رفتار سالم میباشد که معمولا به زمان زیادی نیاز دارد. همانگونه که در بازاریابی تجاری برای تشویق خریدار محصول باید جذاب نشان داده شود، در بازاریابی اجتماعی هم محصول که همان رفتار مطلوب اجتماعی است باید به گونه ای جذاب نشان داده شود تا خود مصرف کنندگان تمایل داشته باشند به طور "خود مختار" وارد فرآیند تغییر رفتار شوند.

درواقع در بازاریابی اجتماعی از رسانه های جمعی، اجتماعی و الکترونیکی و آموزش های جذاب صرفاً برای متقاعد کردن مردم برای انجام رفتار مطلوب اجتماعی استفاده نمی شود بلکه هدف بر این است تا انجام این رفتار تبدیل به گزینه مطلوب مردم شده و سپس به صورت "قاعده" دربیاید. تفاوت بازاریابی اجتماعی با سایر مدل ها از همین جا شروع میشود. در بازاریابی اجتماعی این خود مردم هستند که رفتار صحیح را انتخاب می کنند ولی در سایر مدل ها به نگاه از بالا به پایین به نوعی رفتار مطلوب به جامعه تحمیل میشود. به عبارت دیگر بازاریابی اجتماعی حالت مشتری مدار داشته و با در نظرگرفتن نیازهای حوزه سلامت گروه های مختلف جامعه هدف، سعی در برآورده کردن آن نیازها و در نتیجه تشویق مردم به استفاده از رفتار مطلوب می شود.(1, 2)

در بازاریابی تجاری هدف منفعت فروشنده است در حالی که در بازاریابی اجتماعی هدف منفعت مشتری یا جامعه است.(3)

لازم به ذکر است که بازاریابی اجتماعی با تبلیغات نباید اشتباه گرفته شود هرچند تبلیغات یکی از ابزارهای مهم بازاریابی اجتماعی است. در بازاریابی اجتماعی علاوه بر تبلیغات باید جامعه هدف مشخص باشد، نیازهای این جامعه هدف را در نظر گرفته شده و موانعی که دسترسی به رفتار مورد نظر را دشوار می کند برطرف کرد و دستیابی به رفتار را تسهیل نمود و پس از دستیابی به رفتار مورد نظر با حمایت بیشتر از عقب گرد به حالت اول جلوگیری نمود.(3)

مرکز ملی بازاریابی اجتماعی انگلستان (National Social Marketing Centre) بازاریابی اجتماعی را اینگونه تعریف میکند: دیدگاهی که موجب تکامل فعالیت هایی با هدف تغییر یا حفظ رفتار مردم به سود اشخاص یا جامعه به عنوان یک کل میشود.(4) از این تعریف سه نکته برداشت میشود:

1. بازاریابی اجتماعی یک فرآیند برنامه ریزی شده و سیستماتیک است.(که طبق دستورالعمل این سازمان شامل شش مرحله آغاز به کار، تعیین جامعه هدف، تکامل استراتژی، اجرای طرح، ارزیابی و پیگیری میباشد)
2. سودرسانی به مردم و جامعه توسط ارزشی که توسط خود مردم ارزشگذاری میشود تعیین میشود نه چیزی که سازمان های مختلف فکر میکنند برای مردم مفید است.
3. هدف بازاریابی اجتماعی همواره تغییر یا حفظ رفتار است نه صرفاً آگاهی دادن به مردم

متدهایی که در بازاریابی اجتماعی استفاده میشوند مانند متدهایی است که در بازاریابی تجاری استفاده میشوند مانند:

1. Exchange Theory: به رابطه بین هزینه یک شی و ارزشی که اشخاص برای آن قائل هستند میپردازد. برای اینکه بازاریابی اجتماعی موفق باشد باید رفتار مطلوبی که به افراد "فروخته" میشود دارای ارزش بیشتری از رفتار رقیب که همان رفتار ناسالم است داشته باشد. در بازاریابی اجتماعی ما به دنبال این هستیم که بفهمیم چرا یک رفتار ناسالم دارای جذابیت بیشتری نسبت به رفتار سالم در نزد افراد دارد.
2. Audience segmentation(تقسیم بندی مخاطبان): در این میان بدیهی است که گروه های مختلف جامعه ارزشهای متفاوتی برای رفتارهای متفاوت قائل میشوند پس تقسیم بندی مخاطبان امری اجتناب ناپذیر است.جمعیت هدف بر اساس ویژگیهای اقتصادی-اجتماعی، دموگرافیک و جغرافیایی تقسیم بندی میشوند.
3. The marketing mix (آمیزه بازاریابی): از چهار P تشکیل شده است:

1. Product: محصول که همان رفتار مطلوب اجتماعی است.

2. Price: هزینه ای است که افراد جهت انجام رفتار سالم باید متحمل شوند.

3. Place: محلی است رفتار در آنجا رخ میدهد.

4. Promotion: چگونه رفتار جدید به گوش مخاطبان رسانده میشود.(استفاده از رسانه های ارتباط جمعی و ...)

4. Consumer orientation(مشتری مداری): اصولی که به ما اطمینان میدهد مداخله ما منطبق با نیازها و ارزشهای هر گروهی از جامعه هدف است.

5. Continuous monitoring(ارزیابی پیوسته): باعث پایش، به روزرسانی و در صورت لزوم اصلاح استراتژی بازاریابی میشود. (5)

برای سازماندهی فعالیت ها در طی بازاریابی اجتماعی میتوان از الگوی social marketing assessment and response tool (SMART) استفاده کرد که شامل هفت مرحله برنامه ريزي مقدماتي، تحليل

گروه هدف، تحليل بازار، تحليل كانال، توليد مواد و پيام ها و پيش آزمون آنها، اجرا و ارزشيابي می باشد و طی کار به ترتیب مراحل آن باید اجرا شود.(6)

برای شروع یک بازاریابی اجتماعی ابتدا لازم است تا مشکل به طور دقیق و بدون ارائه راه حل، مشخص شود. این کار باعث می شود تا راه حل های مختلف امکان بررسی داشته باشند. سپس باید اهداف ما مشخص شود و اینکه ما بعد از بازاریابی به چه چیزی می خواهیم برسیم. سپس گروه بندی مخاطبین را صورت داده و سعی می شود تا دیدگاه این گروه ها نسبت به مشکل به خوبی شناخته شود. برای این کار می توان از مصاحبه یا نظرسنجی ها استفاده کرد. پس از شناخت جامعه هدف باید راهکارهای حل مسئله را ارائه داد و آن ها را به جامعه هدف از طریق تبلیغات یا وسایل دیگر انتقال داد.(3)

به طور کلی این طور با توجه به این مؤلفه ها به نظر می رسد که بازاریابی اجتماعی در حوزه سلامت دهان نیز مانند سایر حوزه های سلامت می تواند نقش مهمی در ترویج رفتارهای سالم بهداشتی داشته باشد.

***

پلاک میکروبی روی سطح دندان بیوفیلمی از رسوبات نرم است که روی سطح دندان و سایر سطوح سخت و نرم حفره دهان میچسبد و به وسیله آب برداشته نمیشود. این تجمع پلاک در ابتدا به وسیله فعل و انفعالات باکتری ها با دندان آغاز شده و سپس از طریق فعل و انفعالات فیزیکی و فیزیولوژیک میان گونه های مختلف باکتریها درون توده میکروبی ادامه پیدا میکند. این پلاک میکروبی دلیل عمده بیماریهای پریودنتال و پوسیدگی دندان و تشکیل جرم میباشد.(7)

رشد پلاک در عرض چند ساعت رخ داده باید حداقل هر 48 ساعت یکبار در افرادی که از نظر پریودنتال سالم هستند کاملا برداشته شود تا از بروز التهاب جلوگیری شود. انجمن دندانپزشکی امریکا (ADA) توصیه می کند که افراد دوباردر روز با مسواک نرم و به مدت حداقل دو دقیقه (30 ثانیه برای هر کوادرانت فکی) مسواک بزنند. روش توصیه شده مسواک زدن هم modified bass می باشد. همچنین نخ دندان یا سایر تمیزکننده های بین دندانی یکبار در روز استفاده شود تا پلاک به طور موثر حذف شود و از ژنژیویت جلوگیری شود. توصیه به دوبار مسواک کردن در روز به این دلیل است که اغلب افراد با یک بار مسواک زدن به اندازه کافی پلاک را برنمیدارند.(8, 9)

با این همه علی رغم اهمیت بالای مسواک زدن، خیلی از افراد جامعه برای انجام دادن آن انگیزه کافی ندارند ولی این احتمال میرود که با استفاده از روش بازاریابی اجتماعی در مقایسه با روشهای آموزشی سنتی معمول و کم هزینه تر مانند پمفلت، بتوان دانش و انگیزه بیشتری در افراد ایجاد نمود.

از آنجایی که در پایان 6 سالگی، کودک می بایست وظیفه رعایت بهداشت دهان و دندانش را خود بر عهده بگیرد و والدین صرفاً نقش نظارتی خواهند داشت لذا با توجه به انطباق این دوران با آغاز مقطع ابتدایی دانش آموزان، انجام مداخلات دهان و دندان در این دوره، فرصت خوبی جهت نهادینه شدن رفتارهای بهداشتی دهان و دندان و تغییر رفتارهای مثبت در افراد را فراهم می نماید. از طرف دیگر، یکی از ویژگیهای بازاریابی اجتماعی، ایجاد تغییر رفتار در گروهای سنی مختلف می باشد که با در نظر گرفتن دانش آموزان مقطع ابتدایی (از 7 تا 12 سال) با سطوح مختلف توانایی، درک و دانش متفاوت، می توان به ارزیابی کارایی رویکرد بازاریابی اجتماعی در جمعیت مذکور با هدف ارتقاء سلامت دهان و دندان پرداخت.

در مطالعه حاضر سعی بر این است که رفتار مسواک زدن از لحاظ تعداد دفعات در روز و همچنین مدت زمانی که هر بار مسواک زدن به طول می انجامد با استفاده از اصول بازاریابی اجتماعی افزایش یابد. فلذا با توجه به اهمیت مسواک زدن در ارتقاء سلامت دهان و دندان و همچنین با توجه به اینکه مطالعه مشابهی در زمینه تغییر رفتار مسواک زدن بر اساس مدل بازاریابی اجتماعی در جمعیت مورد نظر صورت نپذیرفته است ضرورت انجام این مطالعه را آشکار می سازد.

1. **فهرست منابعي كه در بیان مساله استفاده شده است:**

1. Brocklehurst PR, Morris P, Tickle M. Social marketing: an appropriate strategy to reduce oral health inequalities? Int J Health Promot Educ. 2012;50(2):81-91.

2. Cheng H, Kotler P, Lee N. Social marketing for public health : global trends and success stories. Sudbury, Mass.: Jones and Bartlett; 2011.

3. box Ct. Understanding Social Marketing: Encouraging Adoption and Use of Valued Products and Practices 2016 [cited 2016 22 June]. Available from: <http://ctb.ku.edu/en/sustain/social-marketing/overview/main>.

4. Centre TNSM. Behavior change through social marketing 2016 [Available from: <http://www.thensmc.com>.

5. Evans WD, McCormack L. Applying social marketing in health care: communicating evidence to change consumer behavior. Med Decis Making. 2008;28(5):781-92.

6. Thackeray R, Neiger B. Use of Social Marketing to Develop Culturally Innovative Diabetes Interventions. Diabetes Spectr. 2003;16(1):15-20.

7. Newman MG, Takei HH, Carranza FnA. Carranza's clinical periodontology. 11th ed. St. Louis, Mo.: Elsevier/Saunders; 2012. xlv, 825 p. p.

8. ADA.org. Learn More About Toothbrushes 2016 [16 May 2016]. Available from: <http://www.ada.org/en/science-research/ada-seal-of-acceptance/product-category-information/toothbrushes>.

9. ADA.org. Brush Teeth 2016 [16 May 2016]. Available from: <http://www.mouthhealthy.org/en/az-topics/b/brushing-your-teeth>.

10. Mumcu G, Köksal L, Şisman N. An Analysis of Oral Health Campaigns from a Social Marketing Perspective. İLETİ-Ş-İM. 2010.

11. Watson JM, Tomar SL, Dodd V, Logan HL, Choi Y. Effectiveness of a social marketing media campaign to reduce oral cancer racial disparities. J Natl Med Assoc. 2009;101(8):774-82.

12. Gholami M, Pakdaman A, Montazeri A, Jafari A, Virtanen JI. Assessment of periodontal knowledge following a mass media oral health promotion campaign: a population-based study. BMC Oral Health. 2014;14:31.

13. Ganss C, Schlueter N, Preiss S, Klimek J. Tooth brushing habits in uninstructed adults-frequency, technique, duration and force. Clin Oral Investig. 2009;13(2):203-8.

14. ‌رضایی‌پندری ح, ‌کشاورزمحمدی ن. کاربرد رویکرد بازاریابی اجتماعی در حوزه سلامت. فصلنامه علمی-پژوهشی آموزش بهداشت و ارتقای سلامت. 1393;2(2):109-3.

1. **سابقه طرح و بررسي متون:**

علی رغم استفاده زیاد بازاریابی اجتماعی در حوزه های مختلف سلامت استفاده آن در مورد بهداشت دهان و دندان زیاد نبوده است. با این حال مقالاتی که در این زمینه موجود است به شرح زیر است:

در یک مرور سیستماتیک توسط دکتر Mumcoدر کشور ترکیه پویش هایی (campaign) که در زمینه بهداشت دهان و دندان با رویکرد بازاریابی اجتماعی تا سال 2010 به انجام رسیده بوده اند مورد بررسی قرار گرفته اند. در این مطالعه 35 کمپین بر اساس نوع پیام، گروه هدف، هدف اصلی، تاکتیک های ارتباطی و ابزارهای رسانه های گروهی مورد مطالعه قرار گرفتند. هدف عمده این کمپین ها معاینه دهان، افزایش آگاهی مردم نسبت به بهداشت دهان و اعمال پیشگیری کننده از پوسیدگی بود.گروه های هدف شامل کودکان، افراد معلول، والدین ، معلمان و دندانپزشکان بودند. استراتژی ها و تاکتیک هایی که برای رساندن پیام به جمعیت هدف استفاده شده بود عبارت از توزیع مسواک و خمیر دندان رایگان، آموزش چهره به چهره، سمینارهای آموزشی و کارتهای ثبت وضعیت سلامت دهان بودند. در این میان تنها 7 پویش از "ابزار اطلاع رسانی" مانند بیلبوردها، پوستر، بروشور و برنامه های تلویزیونی برای ابلاغ پیام به مردم استفاده کرده بودند که مهمترین آنها بروشور و بیلبورد بود.

در کل نتیجه گیری این مطالعه حاکی از ضروری بودن به کار گیری اصول بازاریابی اجتماعی در زمینه بهداشت دهان و دندان بود.(10)

در مطالعه دیگری توسط دکتر Waston و همکاران در سال 2006 به بررسی موثر بودن بازاریابی اجتماعی در کاهش نابرابری های نژادی در زمینه میزان ابتلا به سرطان دهان پرداختند. در این مطالعه که به روش کوهورت اجرا شد جمعیت شهر مداخله و شهر کنترل قبل و بعد از انجام مداخله (پویش اطلاع رسانی) بررسی شدند. در هر شهر 125 سفیدپوست و 125 سیاه پوست وارد مطالعه شدند. نتیجه این مطالعه این بود که جمعیت شهر مداخله به طور قابل ملاحظه ای افزایش آگاهی نسبت به ضرورت معاینه منظم دهان برای کشف سرطان و تمایل نسبت به این کار را نشان میدادند در حالی در شهر کنترل چنین موضوعی مشاهده نمیشد. همچنین افراد سیاه پوست به میزان بسیار بیشتری از سفیدپوستان افزایش آگاهی نسبت به سرطان دهان و تمایل به معاینه دهان را نشان میدادند. هر چند این مطالعه صرفا از ابزارهای اطلاع رسانی برای انجام مداخله استفاده می کرده است.(11)

در مطالعه دیگری که در ایران توسط دکتر غلامی و همکاران در سال 1390 به اجرا درآمد میزان افزایش آگاهی مردم نسبت به بیماری های پریودنتال بدنبال یک پویش رسانه ای مورد بررسی قرار گرفت. در این مطالعه از جمعیت بالغین 55-18 قبل و بعد از اجرای پویش نظرسنجی شده و نتایج این دو نظرسنجی با هم مقایسه شدند. پویش شامل یک کلیپ انیمیشن بوده که به مدت ده روز در شبکه های تلویزیون رسمی ایران(IRIB) به نمایش درآمده است. نتایج مطالعه نشان میداد که کسانی که کلیپ را دیده بودند نسبت به کسانی که ندیده بودند به طور قابل ملاحظه ای افزایش آگاهی را نشان میدادند (0.61 ± 0.96 نسبت به 0.29 ± 0.8 ، p<0.001).(12)

در مطالعه ای در سال 2008 در آلمان توسط دکتر Ganss و همکاران عادت های مسواک زدن افراد از جمله تعداد دفعات، مدت زمان، میزان نیروی دست و روش مسواک زدن بررسی شدند. در این مطالعه اکثر افراد روزی دو مرتبه با نیروی مناسب مسواک میزدند ولی مدت زمان مسواک زدن حدود 90 ثانیه بود که کمتر از حداقل میزان دو دقیقه بود. روش مسواک زدن استاندارد Modified bass هم مشاهده نشد و بیشتر افراد به صورت چرخشی مسواک می زدند. نکته مهم این مقاله این جا بود که پس از آموزش مسواک زدن صحیح به افراد، تنها 25.2% افراد توانستند تمام معیارهای مسواک زدن صحیح را اجرایی کنند که دلیلی بر ناکارآمد بودن آموزش بر اساس روش های سنتی است.(13)

در یک مطالعه سیستماتیک دیگر که توسط حسن رضایی و دکتر کشاورز اجرا شد به بررسی موثر بودن بازاریابی اجتماعی در حوزه سلامت به طور عمومی نه بخش دهان و دندان پرداخته شده است. در این مطالعه محور عمده مطالعات تغذیه سالم بوده است. در این مطالعات نتیجه بازاریابی اجتماعی نه تنها بر رفتار مصرف یک محصول خاص بلکه بر متغیرهای دیگری همچون آگاهی، باورها و وضعیت سلامت نیز گزارش شده است. میزان موفقیت گزارش شده در تغییر رفتار از 10 تا 30 درصد متفاوت بوده است. نتیجه گیری این مطالعه این بوده است که در مداخلات ارتقاء سلامت با رویکرد بازاریابی اجتماعی در تغییر رفتارهایی که مستلزم استفاده از یک محصول بهداشتی و دسترسی به امکانات است و نیز بیشتر تحت تأثیر نگرش و تصمیم فردی است میتواند با موفقیت های ارزشمندی همراه باشد(14).

1. **فهرست منابعي كه در بررسي متون استفاده شده است:**

10. Mumcu G, Köksal L, Şisman N. An Analysis of Oral Health Campaigns from a Social Marketing Perspective. İLETİ-Ş-İM. 2010.

11. Watson JM, Tomar SL, Dodd V, Logan HL, Choi Y. Effectiveness of a social marketing media campaign to reduce oral cancer racial disparities. J Natl Med Assoc. 2009;101(8):774-82.

12. Gholami M, Pakdaman A, Montazeri A, Jafari A, Virtanen JI. Assessment of periodontal knowledge following a mass media oral health promotion campaign: a population-based study. BMC Oral Health. 2014;14:31.

13. Ganss C, Schlueter N, Preiss S, Klimek J. Tooth brushing habits in uninstructed adults-frequency, technique, duration and force. Clin Oral Invest. 2009;13(2):203-8.

14. ‌رضایی‌پندری ح, ‌کشاورزمحمدی ن. کاربرد رویکرد بازاریابی اجتماعی در حوزه سلامت. فصلنامه علمی-پژوهشی آموزش بهداشت و ارتقای سلامت. 1393;2(2):109-3.

1. **اهداف اصلي (کلی) طرح:**

تعیین تأثیر مداخله آموزشی مبتنی بر بازاریابی اجتماعی بر افزایش دفعات و مدت زمان مسواک زدن خوداظهار دانش آموزان مقطع ابتدایی در شهر کهک استان قم در مقایسه با تأثیر آموزش سنتی (پمفلت) بر افزایش دفعات و مدت زمان مسواک زدن خوداظهار دانش آموزان در همان مقطع تحصیلی در شهر جعفریه استان قم

1. **اهداف اختصاصی (ويژه) طرح:**

اهداف تحلیلی:

- تعیین رابطه بین انجام مداخله آموزشی براساس بازاریابی اجتماعی در مقایسه با روش سنتی (پمفلت) با دفعات مسواک زدن دانش آموزان دبستانی
- تعیین رابطه بین انجام مداخله آموزشی براساس بازاریابی اجتماعی در مقایسه با روش سنتی (پمفلت) با مدت زمان مسواک زدن دانش آموزان دبستانی

1. **اهدف كاربردي طرح:**

بهبود وضعیت بهداشت دهان ودندان با استفاده صحیح و متناوب از مسواک

1. **فرضيات و سوالات پژوهش:**

فرضیات:

- درصد دفعات مسواک زدن توصیه شده در دو روش مداخله آموزشی براساس بازاریابی اجتماعی و روش سنتی (پمفلت) در دانش آموزان دبستانی یکسان است.
- درصد پیروی از مدت زمان مسواک زدن توصیه شده در دو روش مداخله آموزشی براساس بازاریابی اجتماعی و روش سنتی (پمفلت) در دانش آموزان دبستانی یکسان است.

1. **نوع مطالعه:** مطالعه مداخله ای (نیمه تجربی)
2. روش اجرا:

**توصیف مطالعه:**

این مطالعه که به صورت یک مطالعه نیمه تجربی شاهد دار فاز 3 و به صورت موازی طراحی شده است و تأثیر مداخله آموزشی بر اساس بازاریابی اجتماعی در ارتباط با دفعات و مدت زمان مناسب مسواک زدن در میان دانش آموزان ابتدایی در شهر کهک (گروه مداخله) در مقایسه با تأثیر مداخله سنتی و کم هزینه (پمفلت) در میان دانش آموزان ابتدایی شهر جعفریه (گروه شاهد) مورد بررسی قرار میگیرد.

**معیار ورود و خروج افراد:**

دانش آموزان مشغول به تحصیل در کلاس های اول تا ششم ابتدایی مدارس دو شهر کهک و جعفریه در سال تحصیلی 95-96 اجازه ورود به مطالعه را دارند. همچنین عدم همکاری دانش آموز یا عدم رضایت خود یا والدین معیار خروج افراد از مطالعه می باشد.

**مکان و نحوه جمع آوری اطلاعات:**

محل انجام مطالعه در مدارس ابتدایی دخترانه و پسرانه دو شهر کهک و جعفریه استان قم می باشد. در مرحله پیش و پس از مداخله، عملکرد خوداظهار جمعیت مورد مطالعه در ارتباط با دفعات و مدت زمان مسواک زدن توسط چک لیست ( پیوست 1) بررسی می گردد. چک لیست دانش آموزان پایه اول تا سوم در مقطع ابتدایی توسط والدین آنها و مقاطع چهارم تا ششم ابتدایی توسط خودشان تکمیل می گردد.

**مداخله هر گروه:**

مداخله در شهر کهک استفاده از اصول بازاریابی اجتماعی مانند ابزارهای تبلیغاتی، کلاس آموزشی، توزیع مسواک، زمان سنج، بروشور و ... جهت ترویج عادات مطلوب مسواک زدن(دو بار در روز و دو دقیقه) و در شهر جعفریه استفاده از روشی سنتی مانند توزیع پمفلت می باشد.

**نتایج اولیه و ثانویه از پیش مشخص شده:**

نتایج اولیه بهبود تعداد دفعات و مسواک زدن افراد می ‌باشد و نتایج ثانویه ای برای این مطالعه در نظر گرفته نشده است. نتایج اولیه با ثبت خود اظهار در چک لیست به دست می آید.

**نحوه تعیین حجم نمونه:**

-

**روش آنالیز نتایج:**

مقایسه توزیع متغیرهای پیامد کیفی (مانند متغیرهای دفعات توصیه شده مسواک زدن و رعایت مدت زمان مسواک زدن توصیه شده) بین دو گروه مطالعه بر اساس آنالیز Chi-2 و رگرسیون لوجیستیک انجام می شود.

1. **روش آنالیز داده ها**

مقایسه توزیع متغیرهای پیامد کیفی (مانند متغیرهای دفعات توصیه شده مسواک زدن و رعایت مدت زمان مسواک زدن توصیه شده) بین دو گروه مطالعه بر اساس آنالیز Chi-2 انجام می شود.

1. **مشخصات ابزار جمع آوري اطلاعات و نحوه جمع آوري آن:**

توسط چک لیست شاخص های مدت زمان و تعداد دفعات مسواک زدن بررسی میشود. این چک لیست در دانش آموزان کلاس اول تا سوم توسط والدین آنها و در دانش آموزان کلاس چهارم تا ششم توسط خودشان تکمیل میشود.

1. **روش نمونه گیری:**

-

1. **روش محاسبه حجم نمونه و تعدادآن:**

به علت جمعیت کم دانش آموزان دو شهر از نمونه گیری استفاده نخواهد شد و سرشماری تمام جامعه هدف انجام خواهد شد.

1. **ملاحظات اخلاقي:**

مورد اول کسب مجوزهای لازم از آموزش و پرورش و شهرداری منطقه می‌باشد که طی مکاتباتی با این نهاد ها صورت خواهد گرفت.

مورد بعد کسب رضایت افراد شرکت کننده در طرح می باشد که با توجه به جامعه نگر بودن مطالعه در ابتدای هر چک لیست که به دانش آموزان داده می شود قسمتی جهت اخذ رضایت از والدین دانش آموزان گنجانده شده که در صورت رضایت آن را امضا کرده و چک لیست به مجری طرح برگردانده می شود. (پیوست 1)

1. **محدوديتهاي اجرايي طرح وروش كاهش آنها:**
2. مراحل انجام مطالعه شامل مرحله قبل از مداخله، مداخه و پس از مداخله می بایست در طول سال تحصیلی (و ترجیحا یک سال تحصیلی) صورت پذیرد لذا امکان آغاز مطالعه در فصل تابستان وجود نداشته و باید تا آغاز سال تحصیلی صبر کرد.
3. تبلیغات گسترده در سطح مدرسه مداخله جهت بازاریابی اجتماعی نیازمند هزینه زیاد و همکاری مسئولین مدرسه و آموزش و پرورش می باشد که می توان از طریق مراجعات حضوری و تبیین ضرورت انجام مطالعه، همکاری آنان را اخذ نمود.
4. عدم مشارکت افراد شرکت کننده در مطالعه پس از انجام مداخله(مرحله دوم) می‌تواند باعث کاهش کیفیت نتایج شود. البته با توجه به حضور دانش آموزان در مدرسه احتمال این امر کاهش می‌بابد مگر در حالتی که دانش آموز غایب باشد که برای جبران آن میتوان در روز دیگری معاینه انجام شود.
5. **جدول متغيرها :**

| رديف | عنوان متغير | نقش متغير | | نوع متغیر | | | | تعريف علمي - تعریف عملي | نحوه اندازه گيري | مقياس (واحد) |
| --- | --- | --- | --- | --- | --- | --- | --- | --- | --- | --- |
|  |  |  |  | كمي | | كيفي | |  |  |  |
|  |  | مستقل | وابسته | پيوسته | گسسته | اسمي | رتبه‏اي |  |  |  |
|  | مدت زمان مسواک زدن |  | * |  |  | * |  | میانگین زمانی هر بار مسواک زدن دندانها | پرسش از دانش آموز و والدین | مساوی یا بیشتر از 2 دقیقه/ کمتر از 2 دقیقه |
|  | دفعات مسواک زدن |  | * |  |  | * |  | میانگین تعداد دفعات مسواک زدن روزانه | پرسش از دانش آموز و والدین | مساوی یا بیشتر از دو بار در روز/ کمتر از دو بار در روز |
|  | سن | * |  |  | * |  |  | تعداد سالهای زندگی | پرسش از دانش آموز و والدین | سال |
|  | جنس | * |  |  |  | * |  | فنوتیپ ظاهری فرد | پرسش از دانش آموز و والدین | مونث،مذکر |
|  | تحصیلات دانش آموز | * |  |  |  |  | * | میزان سواد بر اساس تعداد سالهای تحصیل رسمی | پرسش از دانش آموز و والدین | پایه اول تا ششم مقطع ابتدایی |
|  | تحصیلات والدین | * |  |  |  |  | * | میزان سواد بر اساس تعداد سالهای تحصیل رسمی | پرسش از دانش آموز و والدین | تعداد سال تحصیل |
|  | مداخله آموزشی | * |  |  |  | * |  | آموزش مدت زمان و دفعات صحیح مسواک زدن | براساس روش مطالعه | روش آموزش با پمفلت/ روش بازاریابی اجتماعی |

1. **جدول زمان بندي مراحل اجراي طرح:**

پيش بيني كل زمان لازم براي اجراي كامل طرح به ماه: 12 ماه

| رديف | فعاليتهاي اجرائي | زمان كل |  |  |  |  |  |  |  |  |  |  |  |  |
| --- | --- | --- | --- | --- | --- | --- | --- | --- | --- | --- | --- | --- | --- | --- |
| 1 | طراحی ابزارهای انتقال پیام و متدهای قابل استفاده در بازاریابی اجتماعی | 2 |  |  |  |  |  |  |  |  |  |  |  |  |
| 2 | کسب مجوز های لازم از آموزش و پرورش و شهرداری | 1 |  |  |  |  |  |  |  |  |  |  |  |  |
| 3 | ارزیابی پیش از مداخله | 2 |  |  |  |  |  |  |  |  |  |  |  |  |
|  | اجرای مداخله | 1 |  |  |  |  |  |  |  |  |  |  |  |  |
|  | ارزیابی پس از مداخله | 2 |  |  |  |  |  |  |  |  |  |  |  |  |
| 4 | مقایسه نتایج و آنالیز آماری | 2 |  |  |  |  |  |  |  |  |  |  |  |  |
|  | تهیه مقاله و ارائه پایان نامه | 2 |  |  |  |  |  |  |  |  |  |  |  |  |

## قسمت سوم – اطلاعات مربوط به هزينه‏ها

1. **هزينه كارمندي (پرسنلي) باذكر مشخصات كامل و ميزان اشتغال هرفرد و حق الزحمه آنها :**

| رديف | نوع فعاليت | نام فرد يا افراد | رتبه علمي | تعداد افراد | كل رقم حق الزحمه براي يك نفر | جمع كل |
| --- | --- | --- | --- | --- | --- | --- |
|  | **طرح دهنده** | **مهدیا غلامی** | **استادیار** | **1** | **h145×48000** | **6،960،000** |
|  | **طرح دهنده** | **احمدرضا شمشیری** | **استادیار** | **1** | **h105×48000** | **5،040،000** |
|  | **مشاور آماری طرح** | **احمدرضا شمشیری** | **استادیار** | **1** | **h105×48000** | **5،040،000** |
|  | **همکار طرح** | **محمد تکمار** | **دانشجو** | **1** | **h130×30000** | **3،900،000** |
| 1. 5 | **همکار طرح** | **رضا رجب زاده** | **دانشجو** | **1** | **h70×30000** | **2،100،000** |
|  |  |  |  |  |  | **23،040،000** |

1. **هزينه آزمايشها وخدمات تخصصي كه توسط دانشگاه ويا ديگر موسسات صورت مي گيرد:**

| موضوع آزمايش يا خدمات تخصصي | مركزسرويس دهنده | تعداد كل دفعات آزمايش | هزينه براي هر دفعه آزمايش یا خدمت | جمع ( ريال ) | |
| --- | --- | --- | --- | --- | --- |
| **معاینه دهان و دندان** | **دانشجوی دندانپزشکی** | **372** | **50،000** | **18،600،000** | |
| **برگزاری کارگاههای آموزش سلامت دهان** | **دانشجوی دندانپزشکی** | **4** | **500،000** | **2،000،000** | |
| **جمع هزينه هاي آزمايشها 20،600،000** | | | | | |

فهرست وسايل و موادي كه بايد از اعتبار اين طرح از داخل يا خارج كشور خريداري شود:

1. **وسايل غيرمصرفي:**

| نام دستگاه | كشورسازنده | شركت سازنده | شركت فروشنده ايراني | تعداد لازم | قيمت واحد | **قيمت كل** |
| --- | --- | --- | --- | --- | --- | --- |
| **هد لامپ** | **چین** |  | **شرکت تعاونی دندانپزشکان ایران** | **1** | **1،500،000** | **1،500،000** |

1. **موادمصرفي:**

| **نام ماده** | **كشورسازنده** | **شركت سازنده** | **شركت فروشنده ايراني** | **تعداديامقدار لازم** | | **قيمت واحد** | | **قيمت كل** |
| --- | --- | --- | --- | --- | --- | --- | --- | --- |
| **بسته یکبار مصرف معاینه (شامل سینی، آینه، آبسلانگ، گاز، ماسک، دستکش، سوند)** | **ایران** | **شرکت فیض طب** | **شرکت تعاونی دندانپزشکان ایران** | | **372** | **60،000** | | **22،320،000** |
| **مسواک دارای لوگوی طرح برای گروه مداخله** | **ایران** | **ایده آل ماکو** | **شرکت تعاونی دندانپزشکان ایران** | | **550** | **50،000** | | **27،500،000** |
| **جمع هزینه مواد مصرفی و غیر مصرفی:** | | | | | | | **51320000** | |

1. **هزينه مسافرت :**

| مقصد | تعداد مسافرت در مدت اجراي طرح و منظور آن | نوع وسيله نقليه | تعداد افراد | هزينه به ريال |
| --- | --- | --- | --- | --- |
| **مدارس ابتدایی شهر کهک** | **28 سفر- ثبت شاخص و توزیع و جمع آوری چک لیست در مرحله پیش و پس از مداخله** | **تاکسی تلفنی** | **1** | **1،400،000** |
| **مدارس ابتدایی شهر جعفریه** | **64 سفر- ثبت شاخص و توزیع و جمع آوری چک لیست در مرحله پیش و پس از مداخله** | **تاکسی تلفنی** | **1** | **3،200،000** |
| **مدارس ابتدایی شهر کهک** | **1 سفر- بازدید مدیر و مشاور طرح از فیلد اجرای طرح** | **تاکسی تلفنی** | **2** | **1،000،000** |
| **جمع هزينه هاي مسافرت** | | | | **5،600،000ريال** |

1. **هزينه هاي ديگر**

| **تکثیر چک لیست و رضایتنامه** | **3،000،000 ریال** |
| --- | --- |
| **هزینه تهیه ابزار اطلاع رسانی شامل بنر، پوستر، پمفلت و پلاکارد** | **40،000،000 ریال** |
| **جمع کل** | **43،000،000 ریال** |

1. **جمع هزينه هاي طرح :**

| هزينه پرسنلي | **23،040،000** ريال | هزينه مسافرت | **5،600،000 ريال** |
| --- | --- | --- | --- |
| هزينه آزمايشها و خدمات تخصصي | **20،600،00**ريال | هزينه هاي ديگر | **43،000،000**  ريال |
| هزينه مواد و وسايل مصرفي | **49،820،000 ريال** |  |  |
| هزينه وسايل غير مصرفي | **1،500،000 ريال** | جمع كل | 143،560،000ريال |

بدينوسيله صحت مطالب مندرج در پيش نويس را تائيد مي نمايد و اعلام مي داردكه اين تحقيق به صورت:

1. يك طرح تحقيقاتي در دانشگاه علوم پزشكي تهران و در قالب پايان نامه ارائه شده است.

دکتر مهدیا غلامی
 امضاي مجري طرح

**پیوست 1: چک لیست جمع آوری اطلاعات و رضایت از والدین (مخصوص کلاس چهارم تا ششم)**

والدین گرامی

با سلام

بدینوسیله از شما برای مشارکت در طرح پژوهشی حاضر با عنوان "**مداخله آموزشی جهت بهبود عادات مسواک زدن دانش آموزان مقطع ابتدایی استان قم در سال تحصیلی 96-1395"** دعوت بعمل می آید.

قابل ذکر است دانشکده دندانپزشکی دانشگاه علوم پزشکی تهران با همکاری آموزش و پرورش منطقه اقدام به برگزاری دوره آموزشی در سطح مدرسه جهت بهبود عادات مسواک زدن دانش آموزان نموده است. شرکت فرزند شما در اين مطالعه داوطلبانه است و نتايج تحقيق به صورت محرمانه نزد محققين حفظ شده و بدون ذكر اسامى پاسخ دهندگان و صرفاً از نظر علمى به صورت کلی و گروهی بكار گرفته خواهد شد. نحوه همکاری فرزند شما به صورت پاسخ دهی به چک لیست زیر در دو نوبت با فاصله زمانی حدود یک تا دوماه می باشد.

انتظار میرود پس از انجام این مداخله تعداد دفعات و مدت زمان مسواک زدن دانش آموزان به طرز محسوسی افزایش یابد.

در صورت نیاز می توانید سوالات احتمالی خود را در مورد پژوهش حاضر در تمام مراحل آن از یکی از اعضای تيم پژوهش به نام آقای علی اصغر حبیبی به شماره تماس 09196634589 بپرسيد.

آدرس رابط: دانشکده دندان پزشکی دانشگاه علوم پزشکی تهران ، تهران، انتهای کارگر شمالی، بالاتر از انرژی اتمی

در صورتی که از اهداف این طرح آگاهی یافته اید و برای شرکت فرزندتان در این پژوهش رضایت کامل دارید، لطفآ به سوالات زیر در ارتباط با فرزندتان بطور کامل پاسخ دهید.

**اطلاعات مدرسه**

**نام دبستان**:................................ **کلاس**:.................... **شهر**:.................

**اطلاعات دموگرافیک**

**جنسیت**:1 پسر 2 دختر **تاریخ تولد**: / / 13

**میزان تحصیلات پدر**: 1 بیسواد یا ابتدایی 2 راهنمایی و دیپلم ناقص 3 دیپلم 4 فوق دیپلم و لیسانس

5 فوق لیسانس و بالاتر

**میزان تحصیلات مادر:**  1- بیسواد یا ابتدایی 2- راهنمایی و دیپلم ناقص 3- دیپلم 4- فوق دیپلم و لیسانس

5- فوق لیسانس و بالاتر

***لطفاً این قسمت توسط دانش آموز تکمیل شود:***

**عادات مسواک زدن**

**مسواک زدن دندانهای شما توسط چه کسی انجام می شود؟**

1-خودم مسواک می زنم با نظارت والدین 2- خودم مسواک می زنم بدون نظارت والدین 3- پدر یا مادر برایم مسواک می زند 4- اصلآ مسواک نمی زنم

**شما معمولآ چند بار مسواک می زنید؟**

1- سه بار در روز و بیشتر 2- دو بار در روز 3- یکبار در روز 4- چند بار در هفته 5- یکبار در هفته

6- دو تا سه بار در ماه و کمتر 7- اصلآ مسواک نمی زنم

**هر بار مسواک زدن دندانهای شما معمولآ چقدر طول می کشد؟**

❑1- کمتر از 30 ثانیه ❑ 2- بین 30 ثانیه تا یک دقیقه ❑3- بین یک تا دو دقیقه ❑4- بیشتر از دو دقیقه ❑5- دو تا سه دقیقه ❑6- بیشتر از سه دقیقه

**با احترام فراوان، مجری پژوهشپیوست2: چک لیست جمع آوری اطلاعات و رضایت از والدین (مخصوص کلاس اول تا سوم)**

والدین گرامی

با سلام

بدینوسیله از شما برای مشارکت در طرح پژوهشی حاضر با عنوان "**مداخله آموزشی جهت بهبود عادات مسواک زدن دانش آموزان مقطع ابتدایی استان قم در سال تحصیلی 96-1395"** دعوت بعمل می آید.

قابل ذکر است دانشکده دندانپزشکی دانشگاه علوم پزشکی تهران با همکاری آموزش و پرورش منطقه اقدام به برگزاری دوره آموزشی در سطح مدرسه جهت بهبود عادات مسواک زدن دانش آموزان نموده است. شرکت فرزند شما در اين مطالعه داوطلبانه است و نتايج تحقيق به صورت محرمانه نزد محققين حفظ شده و بدون ذكر اسامى پاسخ دهندگان و صرفاً از نظر علمى به صورت کلی و گروهی بكار گرفته خواهد شد. نحوه همکاری فرزند شما به صورت پاسخ دهی به چک لیست زیر در دو نوبت با فاصله زمانی حدود یک تا دوماه می باشد.

انتظار میرود پس از انجام این مداخله تعداد دفعات و مدت زمان مسواک زدن دانش آموزان به طرز محسوسی افزایش یابد.

در صورت نیاز می توانید سوالات احتمالی خود را در مورد پژوهش حاضر در تمام مراحل آن از یکی از اعضای تيم پژوهش به نام آقای علی اصغر حبیبی به شماره تماس 09196634589 بپرسيد.

آدرس رابط: دانشکده دندان پزشکی دانشگاه علوم پزشکی تهران ، تهران، انتهای کارگر شمالی، بالاتر از انرژی اتمی

در صورتی که از اهداف این طرح آگاهی یافته اید و برای شرکت فرزندتان در این پژوهش رضایت کامل دارید، لطفآ به سوالات زیر در ارتباط با فرزندتان بطور کامل پاسخ دهید.

**اطلاعات مدرسه**

**نام دبستان**:................................ **کلاس**:.................... **شهر**:.................

**اطلاعات دموگرافیک**

**جنسیت**:1 پسر 2 دختر **تاریخ تولد**: / / 13

**میزان تحصیلات پدر**: 1 بیسواد یا ابتدایی 2 راهنمایی و دیپلم ناقص 3 دیپلم 4 فوق دیپلم و لیسانس

5 فوق لیسانس و بالاتر

**میزان تحصیلات مادر:**  1- بیسواد یا ابتدایی 2- راهنمایی و دیپلم ناقص 3- دیپلم 4- فوق دیپلم و لیسانس

5- فوق لیسانس و بالاتر

***لطفاً این قسمت نیز توسط والدین تکمیل شود:***

**عادات مسواک زدن**

**مسواک زدن دندانهای فرزند شما توسط چه کسی انجام می شود؟**

1-خودش مسواک می زند با نظارت والدین 2- خودش مسواک می زند بدون نظارت والدین 3- پدر یا مادر برایش مسواک می زند

4- اصلآ مسواک نمی زند

**فرزند شما معمولآ چند بار مسواک می زند؟**

1- سه بار در روز و بیشتر 2- دو بار در روز 3- یکبار در روز 4- چند بار در هفته 5- یکبار در هفته

6- دو تا سه بار در ماه و کمتر 7- اصلآ مسواک نمی زند

**هر بار مسواک زدن دندانهای فرزند شما معمولآ چقدر طول می کشد؟**

❑1- کمتر از 30 ثانیه ❑ 2- بین 30 ثانیه تا یک دقیقه ❑3- بین یک تا دو دقیقه ❑4- بیشتر از دو دقیقه ❑5- دو تا سه دقیقه ❑6- بیشتر از سه دقیقه

**با احترام فراوان، مجری پژوهش**
